# LidarFooler: A Physical Backdoor Attack Method for LiDAR Point Cloud Classification

**DOI:** 10.3390/s26216722

**Published:** 2026-10-23

**Authors:** Wei Wang, Shaofei Yang, Jiawei Zhu, Yanze Ye, Yang Bai, Jinyin Chen

**Affiliations:** 1 National Key Laboratory of Electromagnetic Space Security, Jiaxing 314000, China; wwzwh@163.com (W.W.); z_jw_1212@163.com (J.Z.); 2 College of Computer Science and Technology, Zhejiang University of Technology, Hangzhou 310023, China; ujiang666888@163.com (Y.Y.); 211124030094@zjut.edu.cn (Y.B.); chenjinyin@zjut.edu.cn (J.C.)

**Keywords:** autonomous driving, light detection and ranging (LiDAR), physical backdoor attack, point-cloud classification, sensor security

## Abstract

Light detection and ranging (LiDAR) point-cloud classifiers are vulnerable to backdoor poisoning, while physical sensing can disrupt triggers designed in the digital domain. We propose LidarFooler, a physical backdoor attack that uses a global trigger composed of multiple spatially distributed local trigger components to establish an association between the complete trigger pattern and an attacker-specified target class. Trigger centers are optimized using classification, geometric-preservation, and dispersion constraints, while expectation-over-transformation augmentation is employed to simulate physical variations during poisoned-sample generation. Extensive experiments on benchmark and physical-world datasets demonstrate the effectiveness and robustness of LidarFooler across different point-cloud classification settings. On digital data, LidarFooler achieves an average clean accuracy of 0.92 and an attack success rate of 0.93. Physical experiments with a Livox Mid-40 sensor achieve corresponding values of 0.93 and 0.86, respectively, with a mean attack success improvement of 42.5 percentage points over IRBA. Further evaluations demonstrate the robustness of LidarFooler under different trigger configurations, poisoning rates, target classes, model architectures, augmentations, and defense strategies. These results reveal a practical physical backdoor vulnerability in LiDAR point-cloud classification and highlight the need for security evaluation and defenses that explicitly account for physical sensing conditions.

## 1. Introduction

Light detection and ranging (LiDAR) is widely used in autonomous driving, robotics, and remote sensing to capture the three-dimensional structure of surrounding objects [1,2,3,4]. Deep neural networks (DNNs) process these point clouds to support classification, segmentation, and navigation. As their predictions inform downstream planning and control, reliable point-cloud perception is essential for the safe operation of these systems.

However, learned perception depends on substantial labeled data, whose collection and annotation are costly. Public datasets such as ModelNet [5] and ShapeNetPart [6] support model development, but reliance on third-party data creates opportunities for backdoor poisoning. An attacker can insert triggered training samples so that the model retains normal accuracy on clean inputs while predicting an attacker-specified class when the trigger appears [7,8,9,10]. This behavior can compromise perception without an obvious decline in normal performance. Recent work has also demonstrated imperceptible backdoor triggers in self-supervised visual representation learning [11], illustrating the continued development of stealthy poisoning mechanisms across perception tasks.

**Figure 1:**
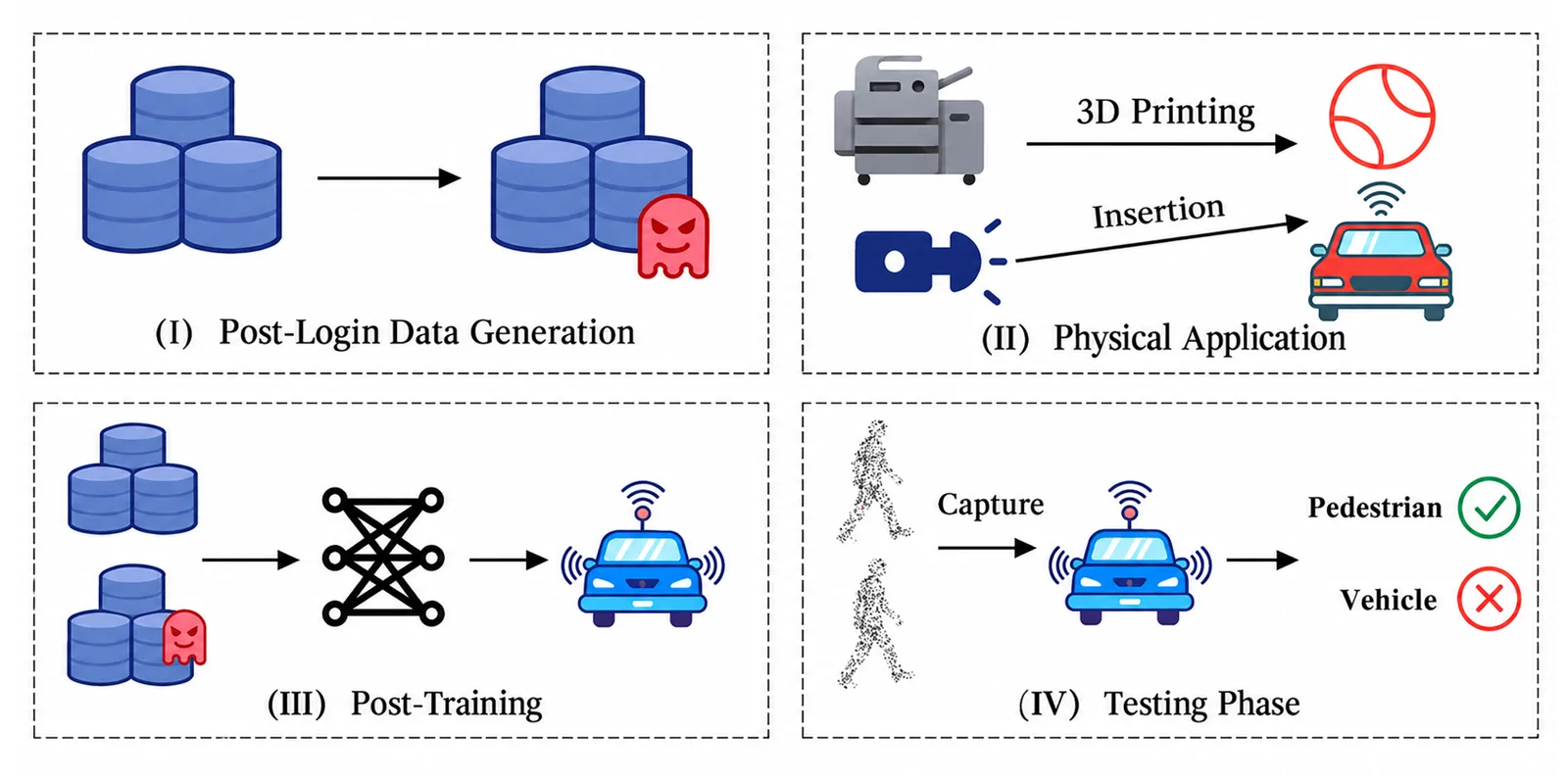
Physical backdoor attack pipeline for LiDAR-based systems.

Physical backdoors additionally require a sensor-captured trigger to activate the poisoned model, as illustrated in Figure 1. Recent work on LiDAR-based object detection shows that physical trigger materials and the gap between digital simulation and sensor measurements affect backdoor activation [12]. Sensor noise, viewpoint changes, distance-dependent sampling, occlusion, and preprocessing can alter the observed pattern. This detection study motivates attention to physical sensing, although the present paper evaluates point-cloud classification. Section 2 reviews existing point-cloud and physical backdoor attacks, while Section 3 formalizes the problem and specifies the threat model.

These limitations pose three challenges: preserving object geometry while inducing the target prediction, maintaining activation under incomplete and perturbed measurements, and supporting different victim architectures when the attacker lacks model knowledge. Recent attacks against 3D scene models and point-cloud classifiers also highlight the importance of plausible object geometry and transfer across models [13,14]. A unified adversarial and backdoor attack on point-cloud classifiers has likewise examined the trade-off between attack effectiveness and geometric stealth [15]. Because preprocessing and occlusion may remove individual trigger components, activation should not depend on a single local region; meanwhile, the injected pattern should avoid conspicuous changes to point-cloud shape and density. Addressing these challenges requires evaluating both clean accuracy and attack success under physical sensing conditions; digital-domain performance alone is insufficient.

We propose LidarFooler to address these challenges using a global trigger composed of multiple spatially separated local trigger components. Throughout this paper, the global trigger denotes the complete distributed trigger pattern, whereas a local trigger component denotes one constituent part of that pattern. Classification, geometric-preservation, and center-dispersion losses optimize the component locations to balance attack effectiveness, limited geometric distortion, and spatial separation. An expectation-over-transformation strategy tailored to LiDAR (EOTL) augments poisoned samples to improve robustness under physical sensing variations.

The main contributions of this work are as follows:

- We propose LidarFooler, a physical backdoor framework whose global trigger consists of multiple spatially separated local trigger components, to attack LiDAR point-cloud classifiers while preserving clean classification performance.
- We develop a trigger-generation method that jointly optimizes classification, geometric-preservation, and center-dispersion objectives and matches trigger density to the original point cloud, balancing attack effectiveness and geometric stealth.
- We incorporate EOTL-based poisoned-sample augmentation to expose trigger patterns to physical variations before deployment and improve their robustness to perturbed sensor measurements.
- We evaluate LidarFooler on ModelNet10, ModelNet40, ShapeNetPart, and a self-constructed physical dataset across four classifier architectures. Livox Mid-40 experiments achieve a mean attack success rate of 0.86 with a mean clean accuracy of 0.93. Additional experiments assess robustness, cross-model transfer, and loss contributions, with an exploratory evaluation of transfer to part segmentation.

The remainder of this paper is organized as follows. Section 2 reviews related work. Section 3 presents the problem formulation and threat model. Section 4 presents LidarFooler. Section 5 and Section 6 describe the experimental setup and analyze the results, respectively. Section 7 concludes the paper.

## 2. Related Work

We review point-cloud and physical backdoor attacks.

### 2.1. Point-Cloud Backdoor Attacks

Point-cloud security research includes attacks that perturb inputs during inference [16,17,18] and backdoors that establish malicious behavior during training. Broader backdoor research explores warped triggers, hidden-trigger poisoning, and deployment-stage activation [19,20,21]. For point clouds, the unordered representation and geometric preprocessing make trigger construction a distinct design problem.

PCBA inserts a point cluster, optimizes its location with an independently trained surrogate classifier, and selects its local geometry to reduce detection by preprocessing and anomaly detectors [22]. PointBA explores interaction and orientation triggers, using added points or object rotation to encode the backdoor [23]. These studies establish point injection and geometric transformation as two important approaches to point-cloud poisoning.

Subsequent work places greater emphasis on stealth and robustness. IRBA combines weighted local transformations with smooth aggregation to resist outlier removal and data augmentation [24]. IBAPC constructs triggers in the graph spectral domain to improve geometric imperceptibility [25], while iBA uses point-cloud reconstruction to produce sample-specific triggers [26]. Thus, resistance to preprocessing is already an explicit objective of existing attacks. Our focus is on combining spatially distributed trigger components with geometry-aware optimization and physical augmentation, and evaluating their activation in LiDAR-acquired measurements.

### 2.2. Physical Backdoor Attacks

Physical backdoors require the trigger to survive the sensing process rather than appear only in a digitally modified input. Wenger et al. investigate backdoor activation using physical objects [27]. Their later work identifies naturally occurring trigger–class subsets in image datasets, showing that potential physical triggers need not be manually inserted into every training image [28]. These studies motivate evaluating how physical acquisition affects trigger activation.

In multimodal perception, BadFusion targets LiDAR–camera fusion detectors through the camera channel and designs triggers to preserve their influence during fusion [29]. Its attack surface differs from the point-cloud classification setting studied here. For LiDAR triggers, viewpoint, distance-dependent sampling, noise, and occlusion can change the observed point pattern. LidarFooler addresses these conditions through distributed trigger placement and EOTL-based augmentation, with physical evaluation using a Livox Mid-40 sensor. The present work does not establish effectiveness against multimodal fusion systems.

## 3. Preliminaries

This section formalizes the point-cloud classification problem and specifies the threat model assumed throughout our attack analysis.

### 3.1. Problem Formulation

We formulate point-cloud classification as follows. Given a deep-learning model $f_{\theta }\left(\cdot \right)$, an input point cloud $X={\left[p_{1},p_{2},\ldots ,p_{N}\right]}^{T}\in R^{N\times 3}$, where $p_{i}=\left(x_{i},y_{i},z_{i}\right)$, and its ground-truth label y∈{1,2,…,C}, the model produces a score vector over *C* classes. The predicted label is determined by the class index with the maximum output score:

(1) $$\hat{y}=\underset{c\in \{1,2,\ldots ,C\}}{\arg\max}\;{\left[f_{\theta }\left(X\right)\right]}_{c},\;I\left(\hat{y}=y\right)\in \left\{0,1\right\},$$

where $f_{\theta }\left(X\right)\in R^{C}$ denotes the model output over *C* classes, *c* is the class index, ${\left[f_{\theta }\left(X\right)\right]}_{c}$ denotes the output score corresponding to class *c*, $\hat{y}$ is the predicted label, *y* is the ground-truth label, and I(·) denotes the indicator function, which equals 1 when the specified condition is true and 0 otherwise.

In a backdoor attack, poisoned training samples induce an association between a trigger and an attacker-specified target class while preserving normal predictions on clean inputs. Let the trigger be T(·) and the backdoored point cloud be $X_{b}=T\left(X\right)$. The backdoored model $f_{\theta^{bd}}\left(\cdot \right)$ has the following desired behavior on clean and triggered inputs:

(2) $$\left\{\begin{array}{c} {\hat{y}}_{\mathrm{clean}}=\underset{c\in \{1,2,\ldots ,C\}}{\arg\max}\;{\left[f_{\theta^{bd}}\left(X\right)\right]}_{c},\;I\left({\hat{y}}_{\mathrm{clean}}=y\right)\in \left\{0,1\right\},\\ {\hat{y}}_{b}=\underset{c\in \{1,2,\ldots ,C\}}{\arg\max}\;{\left[f_{\theta^{bd}}\left(X_{b}\right)\right]}_{c},\;I\left({\hat{y}}_{b}=y_{t}\right)\in \left\{0,1\right\}. \end{array}\right.$$

where *y* is the ground-truth label, $y_{t}$ is the attacker-specified target label, ${\hat{y}}_{\mathrm{clean}}$ denotes the predicted label for the clean point cloud *X*, and ${\hat{y}}_{b}$ denotes the predicted label for the backdoored point cloud $X_{b}$.

### 3.2. Threat Model

We consider a LiDAR perception pipeline in which a user trains a model using a third-party point-cloud dataset. The attacker poisons part of this dataset with triggered samples and deploys a corresponding physical trigger after the trained model is put into use. The sensor captures the trigger together with the object and passes the resulting point cloud to the victim model.

The attacker has access to the training dataset and can insert poisoned samples with an attacker-specified target label. However, the victim architecture, parameters, and training details are unknown, and the attacker cannot control the victim’s training procedure or modify its weights and intermediate features during inference. A locally trained surrogate guides trigger construction. Control over the deployed system is limited to placing the physical trigger in the sensor’s environment.

The attack objective is to induce the target prediction on triggered objects while retaining high classification accuracy on clean inputs. This work primarily evaluates object classification and additionally explores transfer to part segmentation, where the objective is an incorrect segmentation response. Physical attack success requires the sensor-acquired trigger to activate the backdoor, not merely the digitally constructed point pattern.

## 4. Methodology

### 4.1. Overview

**Figure 2:**
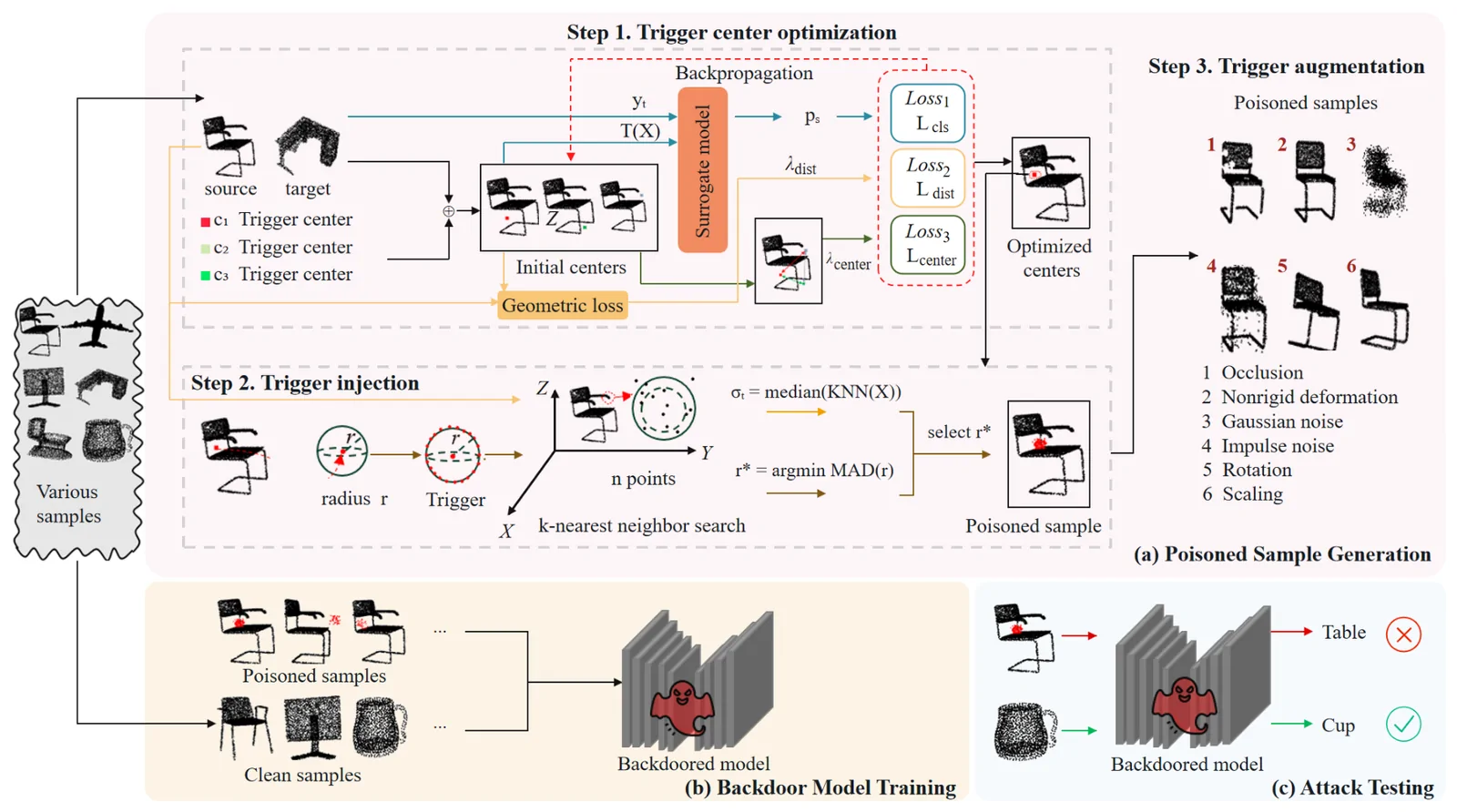
Framework of LidarFooler.

LidarFooler consists of three stages: poisoned-sample generation, backdoored-model training, and attack testing. During poisoned-sample generation, LidarFooler constructs a global trigger composed of multiple spatially separated local trigger components. These components are embedded into the original point cloud through trigger-center optimization and trigger embedding, followed by trigger augmentation to generate poisoned samples. During backdoored-model training, the generated poisoned samples are mixed with clean samples at a specified ratio to construct the backdoor training set, which is then used to train the target model. During attack testing, clean samples and samples carrying physical triggers are separately passed to the backdoored model to evaluate attack effectiveness and stealth. The overall LidarFooler framework is shown in Figure 2.

### 4.2. Trigger Optimization and Poisoned-Sample Generation

LidarFooler constructs backdoored samples in three steps: trigger-center optimization, trigger embedding, and trigger augmentation. First, a surrogate model and multiple loss constraints are used to optimize the trigger-center locations and identify injection positions that jointly satisfy attack effectiveness, spatial dispersion, and physical realizability. Second, the optimized global trigger pattern is instantiated as multiple spatially separated local trigger components, which are embedded at their corresponding positions in the target point cloud to generate poisoned samples. These local components collectively constitute the complete global trigger pattern. Third, a series of augmentations that simulate real-world variations are applied to the poisoned samples to improve trigger adaptability in physical environments. These operations include geometric transformations, noise perturbations, changes in point-cloud density, and nonrigid deformations. They allow the attack to maintain a high success rate under viewpoint changes, partial occlusion, and environmental noise. This design balances attack effectiveness, stealth, and physical robustness.

#### 4.2.1. Trigger Optimization

To effectively deceive a LiDAR-based classification model in the physical domain, a trigger must be both robust and stealthy. Existing backdoor attacks often rely on rigid geometric patterns or high-density point clusters. Such methods can reduce classification accuracy on clean samples and are difficult to implement in real physical environments. We therefore propose a distributed trigger composed of multiple spatially separated local trigger components embedded in the point cloud. Collectively, these local components constitute the global trigger, which denotes the complete distributed trigger pattern used for backdoor activation. When all local components are present, the complete global trigger provides reliable and robust backdoor activation. Under partial visibility or occlusion, only a subset of the local trigger components may be observed; these individual components introduce subtle perturbations while preserving the original geometric structure and thereby improving stealth. The spatially distributed design reduces dependence on any single trigger location and improves robustness to physical transformations and partial occlusion. Overall, the global trigger represents the complete trigger pattern, whereas local trigger components denote its spatially separated constituent parts.

**Trigger optimization.** The injection location is a key determinant of performance in a physical backdoor attack. An inappropriate trigger location may damage the geometric structure of the original point cloud and reduce stealth, while also weakening the trigger’s ability to induce the target class. We first optimize the trigger-center locations to identify injection regions that balance attack effectiveness and geometric naturalness. A surrogate model $f_{\theta }$ is trained on a randomly sampled clean subset $D_{s}={\left\{\left(X_{i},y_{i}\right)\right\}}_{i=1}^{N}$ to approximate the decision behavior of the target model. For the attacker-specified target label $y_{t}$, we define *k* trigger centers $\left\{c_{1},\ldots ,c_{k}\right\}$. To spatially disperse the trigger components and prevent interference caused by overlap, the centers are initialized from a Gaussian distribution $c_{i}∼N\left(0,0.5I\right)$, where *I* denotes the identity matrix. This random initialization enables broad exploration of the three-dimensional space and helps identify trigger locations that provide a high attack success rate without being easily detected. The trigger-point locations must satisfy physical realizability, stealth, and robustness requirements. We therefore define the following loss functions.

**Classification loss.** To ensure that the injected trigger reliably steers the model prediction toward the attacker-specified target class $y_{t}$, we define classification loss $L_{cls}$:

(3) $$L_{cls}=-\log p_{s}\;\left(y_{t}|T\left(X\right)\right)$$

Here, $p_{s}\left(\cdot \right)$ denotes the softmax output of the surrogate model, *X* is the original clean point cloud, and T(X) is the poisoned point cloud after trigger injection. Minimizing this loss increases the model’s confidence in target class $y_{t}$, thereby enabling reliable backdoor implantation.

**Geometric loss.** Optimizing only the classification objective can produce an unnaturally conspicuous trigger distribution and obvious spatial anomalies. To reduce these anomalies, we introduce geometric loss $L_{dist}$:

(4) $$L_{dist}=L_{chamfer}+\lambda_{L2}L_{l2}$$

Here, $L_{chamfer}$ measures the local geometric similarity between the poisoned and original point clouds, and $L_{l2}$ constrains their displacement in the global spatial distribution. Specifically, the Chamfer distance is defined as:   

(5) $$\begin{array}{cc} L_{chamfer}= & \frac{1}{\left|X\right|}\sum_{x\in X}\min_{x^{\prime }\in T\left(X\right)}{∥x-x^{\prime }∥}_{2}^{2}\\ & +\frac{1}{\left|T(X)\right|}\sum_{x\in T(X)}\min_{x^{\prime }\in X}{∥x-x^{\prime }∥}_{2}^{2} \end{array}$$

Here, $L_{chamfer}$ captures the bidirectional nearest-neighbor relationship between the original and poisoned point clouds and thereby constrains local shape changes.

Meanwhile, we define $L_{l2}$ as a squared-L2 proximity loss on the injected trigger points. Let *Q* denote the set of injected trigger points, such that T(X)=X⊕Q. Since adding trigger points changes the point-set cardinality, direct elementwise subtraction between T(X) and *X* is generally undefined. We therefore calculate the mean squared Euclidean distance from each injected point to its nearest point in the original point cloud:

(6) $$L_{l2}=\frac{1}{\left|Q\right|}\sum_{q\in Q}\min_{x\in X}{∥q-x∥}_{2}^{2}.$$

This formulation requires neither equal point counts nor an index-wise correspondence between the original and poisoned point clouds. Minimizing $L_{l2}$ encourages the injected points to remain close to the sampled object geometry. Whereas the bidirectional Chamfer loss evaluates discrepancies between the complete point sets, $L_{l2}$ explicitly averages the proximity penalty over the injected points. These geometric constraints are intended to reduce conspicuous deviations introduced by trigger injection.

**Center loss.** Since the global trigger consists of multiple local trigger components, their centers should remain spatially separated to discourage concentration of the distributed pattern. We therefore introduce an inverse-distance regularizer:

(7) $$L_{center}=\frac{2}{K(K-1)}\sum_{i=1}^{K-1}\sum_{j=i+1}^{K}\frac{1}{{∥c_{i}-c_{j}∥}_{2}^{2}+\varepsilon },\;K\geq 2.$$

Here, *K* is the number of trigger centers, $c_{i}$ and $c_{j}$ are the coordinates of two distinct centers, and ε>0 is a numerical stabilizer that prevents division by zero. The factor 2/[K(K−1)] averages the penalty over all unordered pairs of distinct centers. For K=1, we set $L_{center}=0$.

For each pair of centers, the penalty decreases as their squared distance increases. Consequently, minimizing $L_{center}$ with a positive loss weight discourages closely spaced centers and promotes spatial separation. This regularizer does not guarantee a uniform distribution or complete absence of overlap between trigger components. Instead, it acts jointly with the classification and geometric losses: the center loss discourages clustering, while the proximity loss penalizes trigger points that move far from the original object.

Combining these losses, the final optimization loss $L_{total}$ is defined as:

(8) $$L_{total}=L_{cls}+\lambda_{dist}L_{dist}+\lambda_{center}L_{center}$$

where $\lambda_{dist}$ and $\lambda_{center}$ are hyperparameters that balance the contributions of the loss terms. Jointly minimizing this objective produces optimized trigger centers $C^{*}$ that balance attack effectiveness, stealth, and robustness. Algorithm 1 summarizes the complete trigger-optimization procedure, including spatial initialization, calculation of multiple losses, and iterative gradient updates.
**Algorithm 1** Trigger Center Location Optimization**Require:** Dataset *D*; target label *t*; fixed surrogate model $f_{\theta }$; number of centers *K*; maximum iterations T=1000; learning rate $\eta =10^{-4}$; numerical stabilizer ε>0; iteration limit τ; momentum coefficient μ; loss weights $\lambda_{l2}=0.8$ and $\lambda_{center}=0.5$. **Ensure:** Optimized trigger centers $C^{*}$.
  1:Initialize *K* centers *C* from $N(0,0.5I_{3})$ and set momentum *g* to zero.  2:**for** iter=1,…,min(T,τ) **do**  3:    Sample a point cloud *X* from *D*.  4:    Append *C* to *X* to construct $X_{bd}$.  5:    Compute the target-class cross-entropy loss using $f_{\theta }\left(X_{bd}\right)$ and *t*.  6:    Compute the proximity loss using Equation (6), with the injected point set Q=C.  7:    Compute the bidirectional Chamfer loss between $X_{bd}$ and *X*.  8:    Compute the center-separation loss using Equation (7); set it to zero if K=1.  9:    Combine the losses using the specified weights and unit weight for the Chamfer loss.10:    Compute the gradient of the total loss with respect to *C*.11:    Normalize the gradient with numerical protection and update momentum *g*.12:    Update *C* using *g* and learning rate η.13:**end for**14:**return** $C^{*}\leftarrow C$.


#### 4.2.2. Trigger Injection

After obtaining the optimized trigger centers, we generate a spherical trigger point set containing a total of *n* points, partition it into spatially separated local components, and inject these components into the original point-cloud sample to construct a poisoned sample. To improve physical realizability, we adopt a spherical structure as the basic geometry of the trigger. Specifically, for each candidate radius *r*, the coordinates of the trigger points are generated as

(9) $$\left\{\begin{array}{c} x_{i}=r\sin\theta_{i}\cos\varphi_{i},\\ y_{i}=r\sin\theta_{i}\sin\varphi_{i},\\ z_{i}=r\cos\theta_{i}, \end{array}\right.\;i=1,\ldots ,n,$$

where $\theta_{i}$ and $\varphi_{i}$ are sampled independently from uniform distributions over [0,π] and [0,2π], respectively. An independent random offset sampled from U(−ε,ε) is added to each coordinate of each trigger point. This perturbation introduces slight geometric irregularity and improves the physical realism of the generated trigger.

To match the point spacing of the generated trigger to that of the original point cloud, we employ a K-nearest-neighbor (KNN)-based density-matching procedure. We denote the neighborhood size by $K_{\mathrm{nn}}$ to distinguish it from the number of trigger centers *K*, and set $K_{\mathrm{nn}}=8$. For each point $x_{i}\in X$, its mean KNN distance is calculated as

$$d_{i}^{X}=\frac{1}{K_{\mathrm{nn}}}\sum_{x_{j}\in N_{K_{\mathrm{nn}}}\left(x_{i};X\right)}{∥x_{i}-x_{j}∥}_{2},$$

where $N_{K_{\mathrm{nn}}}\left(x_{i};X\right)$ contains the $K_{\mathrm{nn}}$ nearest neighbors of $x_{i}$ in *X*, excluding $x_{i}$ itself. The reference point-spacing statistic is defined as

(10) $$\sigma_{t}={\mathrm{median}}_{i=1,\ldots ,|X|}\left(d_{i}^{X}\right).$$

The median provides a robust estimate of typical point spacing while reducing the influence of isolated points and extreme local densities.

For each candidate radius *r*, let $P\left(r\right)={\left\{p_{i}\left(r\right)\right\}}_{i=1}^{n}$ denote the generated trigger point set, including the coordinate perturbations. Using the same neighborhood size, we calculate the mean KNN distance of each trigger point within P(r) as

$$d_{i}\left(r\right)=\frac{1}{K_{\mathrm{nn}}}\sum_{p_{j}\left(r\right)\in N_{K_{\mathrm{nn}}}\left(p_{i}\left(r\right);P\left(r\right)\right)}{∥p_{i}\left(r\right)-p_{j}\left(r\right)∥}_{2},$$

where self-neighbors are excluded and $n>K_{\mathrm{nn}}$. The deviation from the reference point spacing is measured by the median absolute deviation from $\sigma_{t}$, denoted here by MAD(r):

(11) $$\mathrm{MAD}\left(r\right)={\mathrm{median}}_{i=1,\ldots ,n}\left(\left|d_{i}\left(r\right)-\sigma_{t}\right|\right).$$

This robust objective reduces the influence of unusually dense or sparse trigger points and encourages the trigger point spacing to match the reference statistic.

Before trigger generation, each input point cloud is centered at its centroid and scaled to fit within the unit sphere. All candidate radii are therefore expressed in dimensionless normalized coordinate units. We set $r_{\min}=0.02$, $r_{\max}=0.20$, and $N_{r}=50$. The candidate radii are uniformly spaced over this interval, including both endpoints:

(12) $$R=\left\{r_{j}=r_{\min}+\frac{j-1}{N_{r}-1}\left(r_{\max}-r_{\min}\right)\;|\;j=1,\ldots ,N_{r}\right\}.$$

Thus, the spacing between adjacent candidate radii is (0.20−0.02)/49≈0.003673. For each input point cloud, the trigger radius is selected as

(13) $$r^{*}=\underset{r\in R}{\arg\min}\mathrm{MAD}\left(r\right).$$

This procedure selects the radius according to the point-spacing characteristics of the input rather than using a single fixed radius for all samples. It is intended to reduce density-related statistical anomalies in the generated trigger.

After selecting $r^{*}$, we partition $P\left(r^{*}\right)=\left\{p_{1},\ldots ,p_{n}\right\}$ into *K* nonoverlapping subsets. Specifically, define $b_{i}=\left\lfloor in/K\right\rfloor$ for i=0,…,K, and let $P_{i}=\left\{p_{j}|b_{i-1}<j\leq b_{i}\right\}$. For example, when K=3 and *n* is divisible by three,

(14) $$\begin{array}{cc} P_{1} & =\{p_{j}|1\leq j\leq n/3\},\\ P_{2} & =\{p_{j}|n/3<j\leq 2n/3\},\\ P_{3} & =\{p_{j}|2n/3<j\leq n\}. \end{array}$$

Each local trigger component $P_{i}$ is translated to its corresponding optimized center $c_{i}$ and merged with the original point cloud to obtain the poisoned sample $X_{b}$:

(15) $$X_{b}=X⊕\overset{K}{\underset{i=1}{⋃}}\left(P_{i}+c_{i}\right),$$

where $P_{i}+c_{i}=\left\{p+c_{i}|p\in P_{i}\right\}$, and ⊕ denotes the operation of combining the trigger points with the original point cloud. The translated local components collectively form the spatially distributed global trigger. Density matching is performed on the complete trigger point set before partitioning and translation; the resulting statistic therefore characterizes the generated spherical point set rather than guaranteeing an exact density match for every translated component.

#### 4.2.3. Trigger Augmentation

Real-world LiDAR point clouds are affected by physical factors such as changes in sensor-to-object distance, viewpoint shifts, partial occlusion, and environmental noise. A trigger constructed only in an idealized digital space may therefore fail to activate reliably after physical deployment. To improve adaptability to real environmental variation, we augment each poisoned sample with multiple physical transformations before training. Inspired by the Expectation over Transformation method, this strategy explicitly simulates different physical perturbations during training so that the model learns a more robust association between the trigger and target label.

Specifically, augmentation function EOTL(·) randomly samples a transformation from a physical-perturbation distribution. For each poisoned sample $X_{b}$, *M* augmented samples are generated:

(16) $$D_{b}^{aug}={\left\{\left(\mathrm{EOTL}\left(X_{b}^{i}\right),y_{t}\right)\right\}}_{i=1}^{M}$$

where EOTL(·) includes four categories of transformations: (1) geometric transformations, including three-dimensional rotation, scaling, and occlusion; (2) noise perturbations, including Gaussian, uniform, impulse, and background noise; (3) point-cloud-density changes, including upsampling, uniform sampling, and density-based sparsification or enhancement; and (4) nonrigid deformations, including spatial warping based on free-form deformation and radial basis functions.

### 4.3. Backdoored Model Training and Attack Evaluation

#### 4.3.1. Backdoored Model Training

After trigger augmentation, all augmented poisoned samples are incorporated into the original training set to train the backdoored model. Let the original training set be $D_{train}$, the augmented poisoned-sample set generated in the previous stage be $D_{b}^{aug}$, and the final backdoor training set be $D_{train}^{\prime }$:

(17) $$D_{train}^{\prime }=D_{train}\cup D_{b}^{aug}$$

Here, every sample in $D_{b}^{aug}$ contains the attacker-designed trigger and is assigned target label $y_{t}$. During training, the model learns not only the classification patterns of normal samples but also a covert association between the trigger pattern and target class, thereby implanting the backdoor behavior.

The target model is optimized using standard supervised learning, with no additional changes to its architecture or training procedure. Using cross-entropy as the loss function, the training objective for the backdoored model is:

(18) $$\theta^{bd}=\underset{\theta }{\arg\min}\sum_{\left(X,y\right)\in D_{train}^{\prime }}L\;\left(f_{\theta }\left(X\right),y\right)$$

Here, $\theta^{bd}$ denotes the trained parameters of the backdoored model. Because the training set retains a large number of clean samples, the model can maintain good classification performance on normal inputs. When a particular trigger is present, however, the model tends to output the attacker-specified target label $y_{t}$. Optimizing the mixed training set $D_{train}^{\prime }$ therefore preserves normal model behavior while implanting a reliable and stealthy backdoor.

#### 4.3.2. Backdoor Activation and Attack Evaluation

After training the backdoored model, we evaluate attack effectiveness during testing. The objective is to compare model behavior on normal and triggered inputs and determine whether the attack is both stealthy and effective. Let the test set be $D_{test}$ and the trained backdoored model be $f_{\theta^{bd}}$. For a clean test sample $\left(X,y\right)\in D_{test}$, the prediction is:

(19) $$y=f_{\theta^{bd}}\left(X\right)$$

When the attacker embeds a physical trigger in an input at test time, the resulting triggered test sample is T(X). The backdoored model’s prediction is:

(20) $$y_{bd}=f_{\theta^{bd}}\left(T\left(X\right)\right)$$

If the attack succeeds, a sample that originally does not belong to the target class is incorrectly predicted as the attacker-specified target label $y_{t}$ after trigger activation, satisfying $y_{bd}=y_{t}$. Evaluation at test time therefore compares the model’s outputs on clean and poisoned samples. Performance on clean samples reflects attack stealth, whereas performance on triggered samples reflects trigger effectiveness. A backdoor attack poses a strong threat in practical settings only when the model maintains high accuracy on clean samples and achieves a high attack success rate on triggered samples.

In summary, LidarFooler implements a physical backdoor attack against LiDAR point-cloud classification models through three stages: poisoned-sample generation, backdoored-model training, and attack testing.

## 5. Experimental Setup

We organize the evaluation around three research questions (RQs), followed by the datasets, target models, evaluation metrics, baseline attacks, and experimental platform.

### 5.1. Research Questions

- **RQ1 (Attack effectiveness):** How effectively does LidarFooler activate the backdoor in digital and physical settings while preserving clean classification accuracy, compared with existing attacks?
- **RQ2 (Robustness and generalizability):** How does attack performance vary with trigger configuration, poisoning rate, target class, model architecture, and perception task, and how well does the attack withstand data augmentation and defenses?
- **RQ3 (Contribution of loss terms):** How do the classification, geometric-preservation, and center-dispersion losses contribute to attack success and geometric stealth?

### 5.2. Datasets

Digital-domain evaluation uses ModelNet10, ModelNet40, and ShapeNetPart, while physical-domain evaluation uses a self-constructed dataset collected with a LiDAR sensor. Together, these datasets support comparisons across object categories and between digital and physical sensing conditions.

ModelNet10 and ModelNet40 provide object-classification benchmarks with 10 and 40 categories, respectively [5]. ModelNet40 contains 12,311 three-dimensional models. Each object is represented as a point cloud with normalized spatial coordinates.

To examine performance on another object collection and explore transfer to part segmentation, we also use ShapeNetPart [6]. It contains 16,846 objects from 16 categories, with annotations for 50 semantic part classes.

For physical evaluation, we use 1000 samples captured with a Livox Mid-40 LiDAR sensor in 10 outdoor and 10 indoor scenes. This dataset is used to assess whether triggers remain effective in sensor-acquired point clouds. Due to limited experimental resources, the physical experiments primarily focused on the chair category, aiming to validate the feasibility of the proposed attack under real-world conditions rather than its generalizability across multiple object categories. Therefore, the reported physical ASR reflects the attack effectiveness within this specific evaluation setting.

### 5.3. Target Models

We select PointNet, PointNet++, DGCNN, and PCT to cover four representative approaches to point-cloud classification: global point-set aggregation, hierarchical local-feature learning, dynamic graph convolution, and attention-based modeling. These architectures provide complementary mechanisms for extracting geometric and semantic features from point clouds and allow us to evaluate the effectiveness of LidarFooler across different model structures.

PointNet extracts pointwise features and aggregates them through symmetric max pooling to process unordered point sets [30]. PointNet++ extends this approach by hierarchically sampling and grouping local neighborhoods, enabling the model to learn geometric features at multiple scales [31]. DGCNN uses EdgeConv to encode relationships between neighboring points and dynamically recomputes the neighborhood graph in feature space [32]. PCT combines neighborhood embedding with attention mechanisms to capture local structure and long-range relationships among points [33]. These architectural differences allow us to examine attack effectiveness across models and evaluate the transferability of LidarFooler between surrogate and victim architectures.

For a fair and reproducible comparison, all models were trained using the same dataset partitioning and point-cloud preprocessing procedures, with a common training protocol. Specifically, Adam was used as the optimizer with an initial learning rate of $1\times 10^{-3}$, a batch size of 32, and 250 training epochs. The learning rate was reduced by a factor of 0.7 when the validation loss did not improve for 20 consecutive epochs, with a minimum learning rate of $1\times 10^{-5}$. A weight decay of $1\times 10^{-4}$ was applied for regularization, and the random seed was fixed to 42 for data partitioning, parameter initialization, and model training. Unless otherwise specified, the resulting trained models were used consistently in the clean classification, backdoor attack, transferability, and defense evaluations.

### 5.4. Evaluation Metrics

We assess attack effectiveness using attack success rate (ASR), the percentage of evaluated triggered test samples classified as the target class:

(21) $$ASR=\frac{\mathrm{Successful}\;\mathrm{attacks}}{\mathrm{Evaluated}\;\mathrm{triggered}\;\mathrm{test}\;\mathrm{samples}}\times 100\%$$

To assess whether the backdoored model preserves normal classification performance, we use clean accuracy (ACC), the percentage of trigger-free test samples classified correctly:

(22) $$ACC=\frac{\mathrm{Correctly}\;\mathrm{classified}\;\mathrm{clean}\;\mathrm{test}\;\mathrm{samples}}{\mathrm{Evaluated}\;\mathrm{clean}\;\mathrm{test}\;\mathrm{samples}}\times 100\%$$

Here, the equations express both metrics as percentages, whereas the tables report the corresponding proportions in [0,1]. Unless otherwise stated, means discussed below are unweighted arithmetic averages of the displayed table entries.

### 5.5. Baselines

We compare LidarFooler with PCBA, PointBA-I, PointBA-O, and IRBA [22,23,24], covering point-injection and geometric-transformation triggers. PointBA-I and PointBA-O denote the interaction and orientation variants, respectively. All four baselines are included in the digital-domain comparison; the physical-domain comparison uses IRBA. The physical implementation and results are described in Section 6.1.

### 5.6. Experimental Platform

Experiments were conducted on a workstation equipped with an Intel Xeon 6240 CPU (2.6 GHz, 18 cores), a Tesla V100 GPU with 32 GiB of GPU memory, and 16 GiB of DDR4-RECC 2666 system memory, running Ubuntu 16.04. The software environment comprised Python 3.7.9, PyTorch 1.10.1, CUDA 11.3, and cuDNN 8.2. The main numerical and point-cloud processing libraries included NumPy 1.21.6, SciPy 1.7.3, and Open3D 0.13.0. Physical experiments used a Livox Mid-40 LiDAR sensor and the associated equipment. All experiments were performed under the same hardware and software configuration unless otherwise specified.

## 6. Experimental Results

### 6.1. Attack Effectiveness

To answer RQ1, we compare attack effectiveness and clean accuracy across the datasets and target models described in Section 5. ASR is used as the principal measure of attack effectiveness. Results on ModelNet and ShapeNetPart are summarized in Table 1 and Table 2. Table 1 reports performance on normal data, and Table 2 reports digital-domain attack results. Results on the self-constructed dataset are reported in Table 3.

Table 2 shows that LidarFooler achieves mean ACC and ASR values of 0.92 and 0.93 across the 12 dataset–model combinations, compared with pooled baseline means of 0.90 and 0.91. These averages do not imply superiority over every individual baseline: PCBA and PointBA-I have higher mean ASR values of 0.95 and 0.96, respectively. On ShapeNetPart, LidarFooler achieves mean ACC and ASR values of 0.97 and 0.96; its mean ACC is higher than those of the baselines, while its mean ASR is slightly lower than those of PCBA and PointBA-I. On ModelNet10 with PCT, its ACC of 0.90 ties PointBA-O, and its ASR of 0.98 ties PointBA-I.

The relative performance varies across settings. On ModelNet10 with DGCNN, PointBA-I and LidarFooler both achieve an ASR of 0.97. On ModelNet40 with PCT, PointBA-I achieves an ASR of 0.96, exceeding LidarFooler’s 0.86. Thus, LidarFooler does not consistently maximize digital-domain ASR. Its distributed triggers and EOTL augmentation are intended to support physical deployment, which we evaluate separately below.

**Figure 3:**
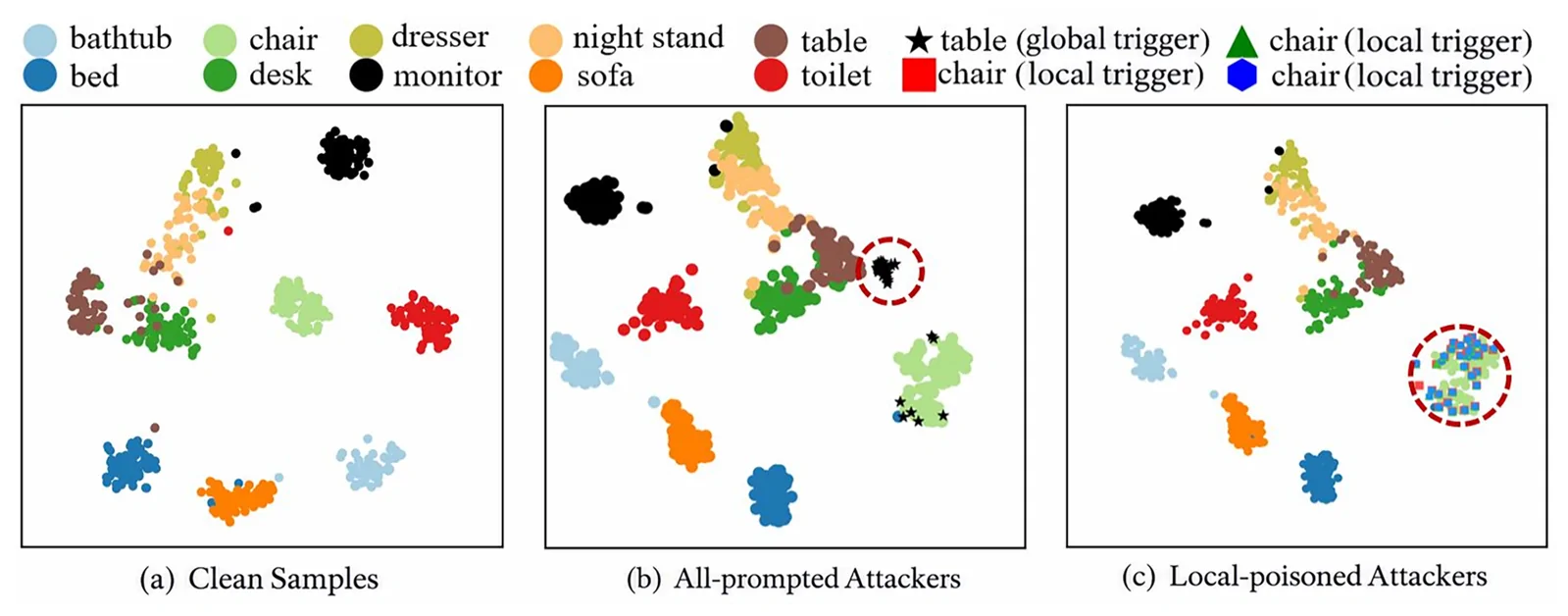
t-SNE visualization of PointNet++ features on ModelNet10.

To further examine how LidarFooler affects the feature space, we visualize PointNet++ features on ModelNet10 using t-SNE, as shown in Figure 3. The left panel shows clean samples, whose features form relatively distinct class clusters. The middle panel shows samples containing the complete global trigger, namely all of its constituent local trigger components. The highlighted triggered-sample features shift toward the target class, “table.” The right panel shows samples containing only a subset of the local trigger components that constitute the same global trigger; the highlighted features remain closer to the original “chair” class. Thus, the comparison concerns the complete distributed pattern and a partially present version of that pattern. These visualizations illustrate differences in feature-space behavior under the two trigger configurations; attack performance is evaluated using the quantitative results reported separately.

In the digital domain, LidarFooler demonstrates a favorable balance between attack success and clean classification performance. Although it does not consistently achieve the highest ASR among all baseline methods, it maintains competitive attack effectiveness while preserving the classification performance of the victim models. These results indicate that LidarFooler provides a practical balance between attack effectiveness and model performance, rather than a universally superior ACC-ASR tradeoff.

**Figure 4:**
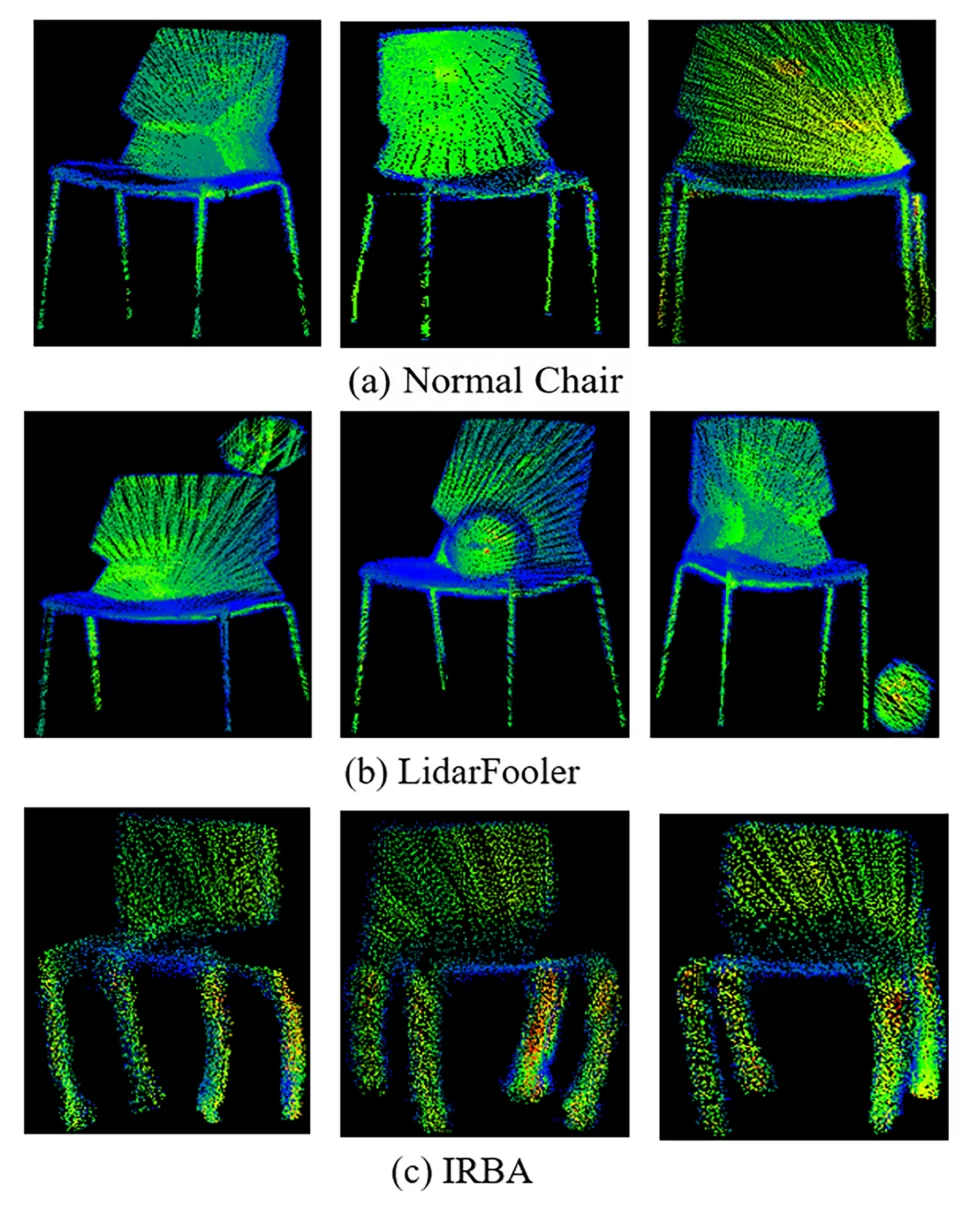
Real-world point clouds captured by a Livox Mid-40 LiDAR sensor.

**Figure 5:**
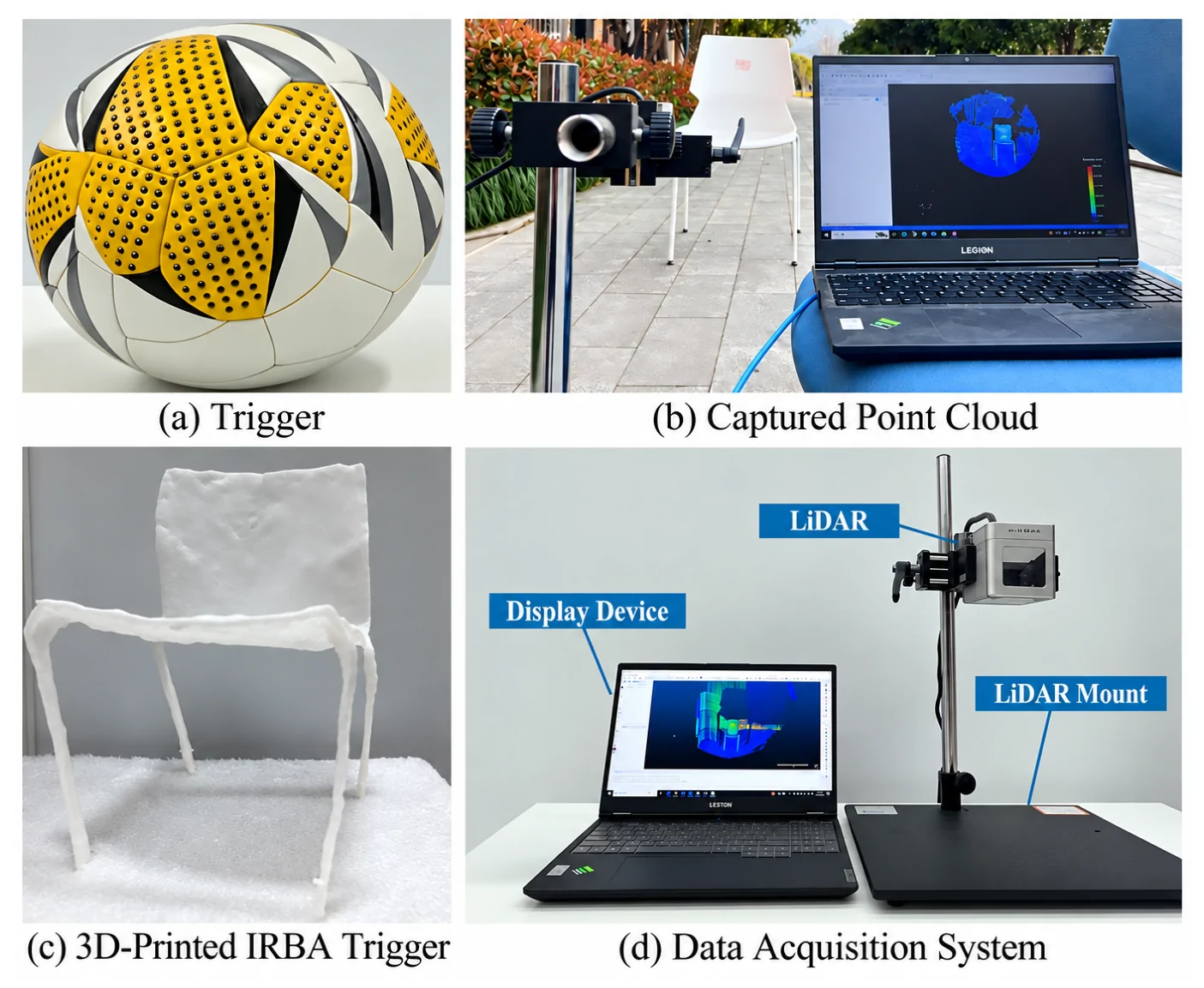
Overview of the physical equipment.

To evaluate LidarFooler in the physical domain, we compare it with IRBA on four models using the self-constructed dataset. The experimental setup is shown in Figure 4 and Figure 5. A Livox Mid-40 LiDAR sensor collects the three-dimensional point clouds. To make physical trigger construction feasible and consistent, all triggers use spherical structures. IRBA is reproduced according to its original settings: backdoored samples are first generated in simulation, physical triggers are then fabricated through three-dimensional printing, and the poisoned model is tested in a real environment. For both methods, triggers are placed at different locations on the target object, a chair, and are successfully activated under all settings. Figure 4 shows real point clouds captured by the Livox Mid-40 in the physical domain, and Table 3 reports the corresponding results.

LidarFooler achieves higher ASR than IRBA on all four models in Table 3. Averaging the displayed table entries gives mean ASR values of 0.8575 and 0.4325, respectively, a difference of 42.5 percentage points. On PointNet++, LidarFooler reaches an ASR of 0.86, compared with 0.73 for IRBA. On DGCNN, the corresponding values are 0.86 and 0.13. LidarFooler’s mean clean ACC is 0.93, although its PCT accuracy of 0.85 is lower than IRBA’s 0.93. These results show improved physical attack success, with clean-accuracy performance varying by model. It should be noted that the datasets and evaluation conditions used for digital experiments differ from those used for physical experiments; therefore, the respective ASR values should be interpreted independently and should not be directly compared.

**Physical Control Experiments.** To further verify that the physical attack success originates from the learned backdoor rather than the presence of additional objects, we conduct two control experiments using the Livox Mid-40 LiDAR.

First, we place the same spherical trigger components in the scene without the optimized spatial arrangement and evaluate their effects on the backdoored models. The number, size, and material of the components remain unchanged, while their positions are selected without trigger optimization. Second, we evaluate the optimized physical trigger on clean models trained without poisoned samples to examine whether the trigger itself acts as an adversarial example. All experiments use consistent physical scenes, acquisition conditions, and point-cloud preprocessing procedures.

As shown in Table 4, the backdoored models achieve an average ASR of 0.86 with the optimized physical trigger, whereas the non-optimized control object yields an average ASR of only 0.09. Moreover, applying the optimized trigger to clean models results in a low average ASR of 0.08. These results suggest that the high physical attack success cannot be explained solely by the presence of an additional object or by the trigger’s inherent adversarial effect. Instead, the substantial difference between the control conditions and the optimized-trigger condition supports the conclusion that physical attack success primarily arises from the learned association between the optimized trigger pattern and the attacker-specified target class.

Two design choices explain this physical-domain improvement. First, unlike the trigger used by IRBA, LidarFooler employs a distributed structure consisting of local and global triggers. This design increases the probability of successful activation when only part of the trigger is visible and therefore maintains attack performance under viewpoint changes or partial occlusion. Second, LidarFooler applies multiple physical transformations during training, including scaling, rotation, and noise. The model consequently learns trigger patterns that remain effective under sensor noise, distance changes, and partial occlusion, substantially narrowing the performance gap between the digital and physical domains.

Overall, LidarFooler achieves higher physical-domain ASR than IRBA across the tested models, with a mean improvement of 42.5 percentage points and a mean clean ACC of 0.93. These results support the effectiveness of the evaluated physical attack configuration.

### 6.2. Robustness and Generalizability

To answer RQ2, we evaluate the robustness and generalizability of LidarFooler from three perspectives. First, we investigate how trigger-design parameters, including the number and shape of triggers and the poisoning rate, affect attack performance. Second, we examine generalization across model architectures, target classes, and tasks. Third, we evaluate stability under representative data-augmentation and defense mechanisms. Together, these experiments provide a broad assessment of LidarFooler in complex settings.

**Sensitivity to trigger configuration.** We evaluate the sensitivity of LidarFooler to the number and shape of local triggers under two dataset-model settings: ModelNet10 with PointNet and ModelNet40 with PointNet++. For each setting, the number of local triggers is varied from one to four, and three trigger shapes, namely circle, rectangle, and triangle, are considered. The results are summarized in Table 5.

For ModelNet10 with PointNet, the mean ACC and ASR across the 12 configurations are 0.915 and 0.900, respectively. The mean ASR increases from approximately 0.86 with one trigger to 0.94 with three triggers, and then decreases slightly to approximately 0.91 with four triggers. A similar trend is observed for ModelNet40 with PointNet++, where the mean ASR increases from approximately 0.78 with one trigger to 0.88 with three triggers, followed by a slight decrease to approximately 0.86 with four triggers. These results indicate that increasing the number of local triggers improves attack performance up to a moderate quantity, while adding more triggers does not necessarily provide further gains. The overall ACC remains relatively stable across the evaluated configurations.

**Figure 6:**
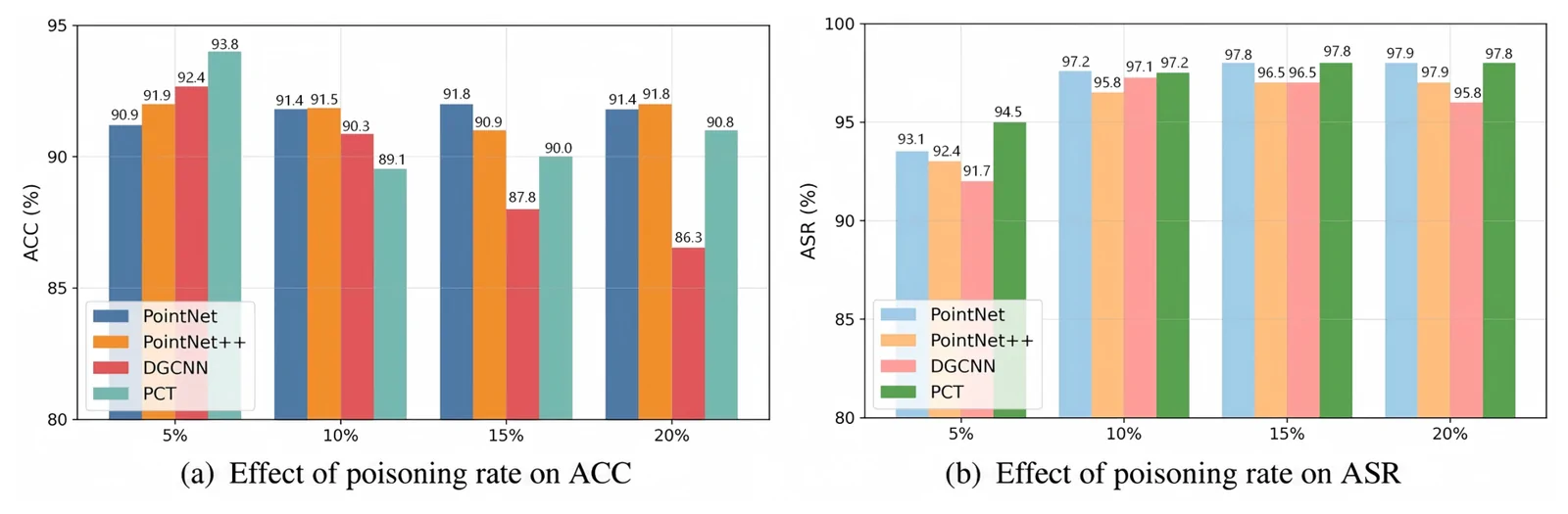
Effect of the poisoning rate on LidarFooler.

Figure 6 shows the effect of poisoning rate. ASR generally increases between 5% and 10%, then remains high with smaller fluctuations at 15% and 20%; it does not increase monotonically for every model. Clean ACC also varies by model: PointNet and PointNet++ remain relatively stable, whereas DGCNN exhibits a more pronounced decrease. The results show a model-dependent tradeoff between poisoning rate, attack success, and clean accuracy.

**Generalization across classes, models, and tasks.** To further examine the generalization of LidarFooler across different target classes, we randomly selected three target labels ($y_{t}=7$, 8, and 9) from each dataset without using any class-specific criterion. The class indices follow the 0-based labeling convention used in our experiments. Accordingly, these target labels correspond to sofa, table, and toilet for ModelNet10; car, chair, and cone for ModelNet40; and knife, lamp, and laptop for ShapeNetPart. For ShapeNetPart, these labels refer to the 16 object-category labels used for classification rather than the 50 part-segmentation labels. The corresponding ACC and ASR results are reported in Table 6. ASR ranges from 0.88 to 0.96 on ModelNet10, from 0.80 to 0.84 on ModelNet40, and from 0.88 to 0.99 on ShapeNetPart. These results demonstrate that LidarFooler remains effective across the randomly selected target classes, although they do not establish its effectiveness for every possible target class.

Table 7 evaluates the cross-model transferability of LidarFooler using different surrogate–victim model combinations. For each surrogate model, the trigger is optimized specifically with that surrogate and then directly transferred to the victim models without further trigger optimization. Across the 16 surrogate–victim combinations, LidarFooler achieves higher ACC than PCBA in 12 cases, the same ACC in three cases, and lower ACC in one case. In terms of the overall results, LidarFooler achieves a mean ACC of 0.91 and a mean ASR of 0.93, compared with 0.87 and 0.96 for PCBA, respectively. Although LidarFooler generally maintains higher clean classification accuracy, its ASR is lower than that of PCBA in 11 of the 16 combinations. The largest ASR gap occurs when DGCNN is used as the surrogate and PointNet++ as the victim, where LidarFooler achieves an ASR of 0.78 compared with 0.95 for PCBA. These results indicate that LidarFooler provides stronger clean-accuracy preservation while maintaining competitive cross-model attack transferability, but does not consistently outperform PCBA in ASR.

**Figure 7:**
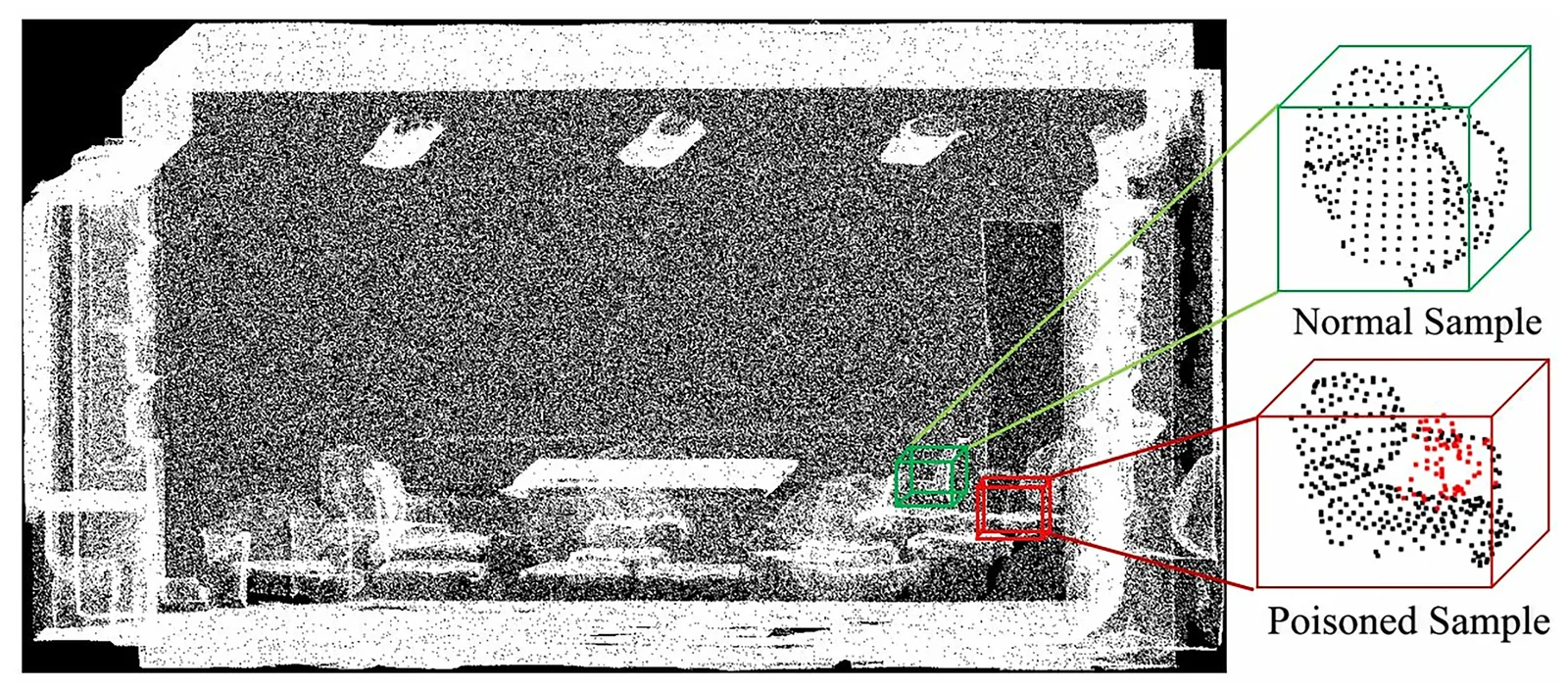
Cross-task transferability of LidarFooler on the Chair category of ShapeNetPart using PointNet++. The normal sample is a clean point cloud without the trigger, whereas the poisoned sample contains the physical trigger. Green and red indicate the normal and poisoned regions, respectively, and the resulting incorrect segmentation demonstrates successful transfer to the segmentation task.

To evaluate cross-task transferability, we directly transfer the physical trigger optimized for classification to three-dimensional point-cloud segmentation without retraining. Specifically, we conduct experiments on the Chair category of ShapeNetPart using PointNet++ for point-cloud part segmentation, as shown in Figure 7. The same physical trigger successfully induces incorrect segmentation predictions, demonstrating the strong cross-task transferability of LidarFooler. The classification and segmentation models generally share similar geometric-feature encoders in their early stages. By perturbing these shared representations through $L_{cls}$, the trigger can induce consistent backdoor activation across different three-dimensional vision tasks.

**Robustness to augmentation, preprocessing, and defenses.** To further evaluate the robustness of LidarFooler under practical processing conditions, we evaluate it against common data-augmentation, preprocessing, and defense methods on ModelNet10 with PointNet as the victim model. The evaluated settings are grouped into three categories: data augmentation, preprocessing, and defense. Specifically, R, R3, scaling, translation, point dropout, and jitter are considered as augmentation settings; SOR is evaluated as a preprocessing method; and DUP and LPF are evaluated as defense methods. Table 8 summarizes the results.

For a fair comparison, all attack methods use the same test split, victim model, evaluation pipeline, and corresponding augmentation operations and intensities. Thus, the observed performance differences reflect the robustness characteristics of the attack-trained models rather than inconsistent evaluation settings. Across the six augmentation settings and SOR, LidarFooler achieves mean ACC and ASR values of approximately 0.89 and 0.90, respectively. Its mean ASR is higher than those of all four baselines, while its mean ACC is slightly below those of PointBA-I and PointBA-O (approximately 0.90).

Notably, substantial differences in clean ACC are observed under rotation. Under R, PCBA and IRBA achieve ACC values of 0.28 and 0.26, respectively, compared with 0.90, 0.90, and 0.91 for PointBA-I, PointBA-O, and LidarFooler. Under R3, the corresponding ACC values are 0.45 and 0.40 for PCBA and IRBA, compared with 0.91, 0.89, and 0.90 for the other three methods. Since the same rotation settings are applied to all methods, these differences reflect the different sensitivities of the attack-trained models to geometric transformations, with LidarFooler maintaining relatively stable clean classification performance.

For SOR, LidarFooler achieves an ACC of 0.82 and an ASR of 0.85. Under DUP and LPF, its ASR values are 0.41 and 0.83, respectively, with a mean of 0.62, while its mean ACC is 0.86. These results indicate that LidarFooler retains effectiveness under the evaluated processing conditions, although its robustness is not uniform across all defenses. This behavior can be partly attributed to the EOTL-based transformations and distributed trigger design, which improve the stability of the learned backdoor representation under geometric and point-level perturbations.

### 6.3. Ablation Study

To answer RQ3, we investigate the contribution of each loss term through an ablation study on ModelNet10 with PointNet as the victim model. Starting from the complete objective, we remove $L_{cls}$, $L_{dist}$, and $L_{center}$ individually while keeping all other settings unchanged. ASR measures attack effectiveness, whereas Chamfer distance (CD) and Wasserstein distance (WD) assess local geometric and global distributional distortion, respectively. All distances reported below follow the displayed scales in Table 9 (CD × 100 and WD × 0.1).

Removing $L_{cls}$ causes the largest decline in ASR, from 0.97 to 0.75, while CD and WD change only slightly to 2.28 and 0.06. This indicates that preserving geometric similarity alone is insufficient to maintain high attack success. The classification loss provides explicit target-label supervision and plays a key role in guiding the backdoor attack toward the attacker-specified target.

Without $L_{dist}$, ASR remains relatively high at 0.93, but CD increases substantially from 2.27 to 3.66, with WD rising to 0.06. Thus, the remaining objectives retain much of the attack effectiveness but allow greater local geometric distortion. This result highlights the importance of $L_{dist}$ in limiting structural changes introduced by trigger injection and preserving geometric stealth.

Removing $L_{center}$ reduces ASR to 0.90 and increases CD and WD to 3.65 and 0.06, respectively. These changes suggest that the center loss contributes to both attack effectiveness and geometric preservation. However, these metrics alone do not establish how this term affects the spatial distribution of trigger centers.

Overall, the complete objective achieves the highest ASR (0.97) and the lowest CD (2.27) and WD (0.05). The ablations highlight the primary contribution of $L_{cls}$ to attack success and the benefits of $L_{dist}$ and $L_{center}$ for geometric preservation. Together, the three terms yield the best measured outcomes, although this study does not separately quantify physical.

**Figure 8:**
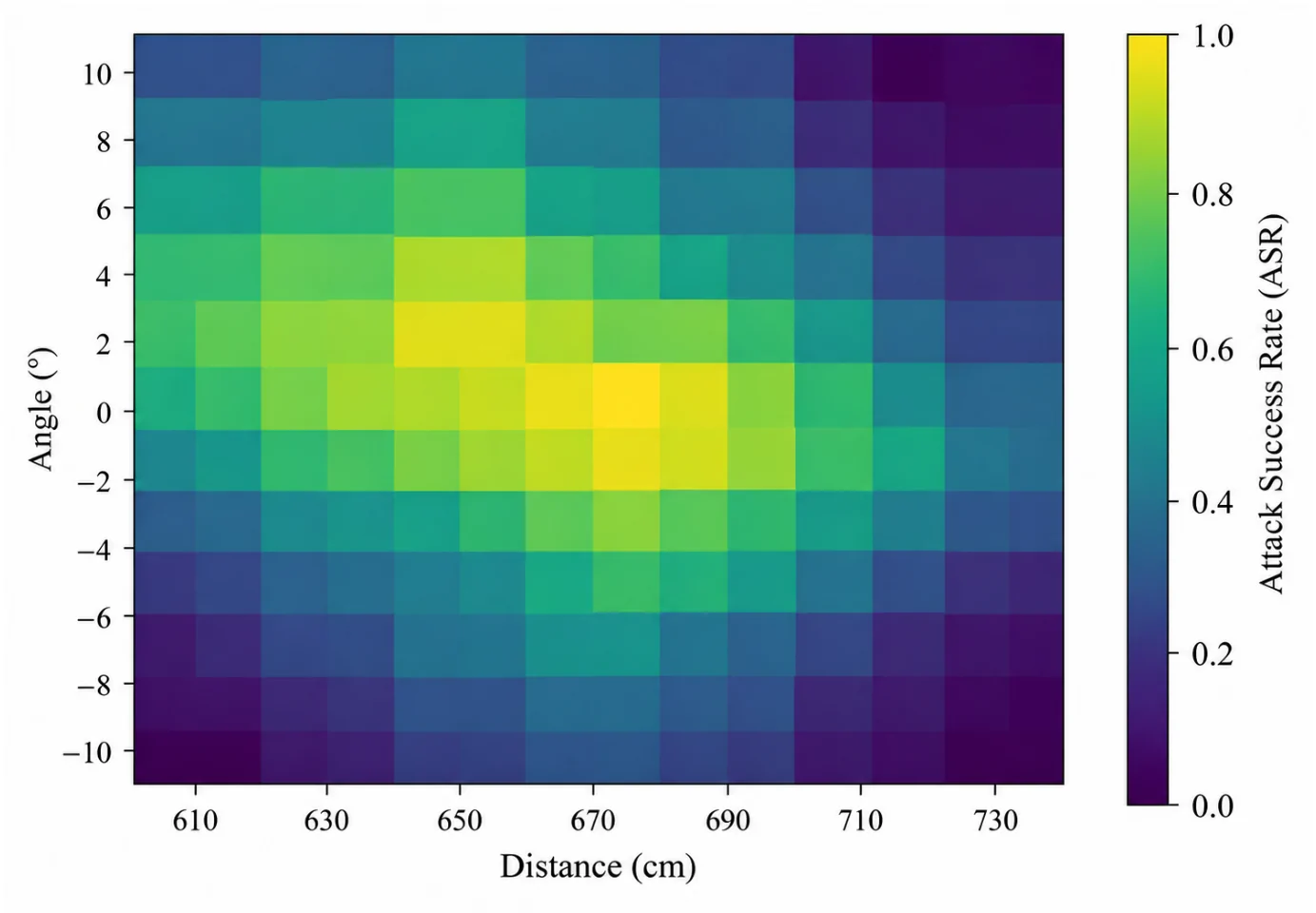
ASR under different sensing distances and viewing angles using the Livox Mid-40 LiDAR.

**Effect of Distance and Viewing Angle.** We further analyzed the sensitivity of the physical attack to sensing distance and viewing angle, considering distances of 6.1–7.3 m and angles of $-10^{\circ }$ to $10^{\circ }$. As shown in Figure 8, the ASR is generally higher at intermediate distances (6.3–6.9 m) and near-frontal viewing angles ($-4^{\circ }$ to $4^{\circ }$), while decreasing toward larger angular deviations and less favorable sensing distances. Notably, the performance exhibits local fluctuations and asymmetric variations, potentially associated with the non-repetitive scanning pattern of the Livox Mid-40 and changes in point-cloud sampling conditions. These observations suggest that sensing geometry may influence attack effectiveness, although further physical measurements are required to validate the simulated trends.

## 7. Conclusions and Future Work

LidarFooler combines spatially distributed triggers with EOTL-based augmentation to enable physical backdoor attacks against LiDAR point cloud classifiers. By jointly considering classification effectiveness, geometric preservation, and trigger point dispersion, it constructs effective triggers under physical sensing variations. Experiments show that LidarFooler achieves an average clean accuracy of 0.92 and attack success rate of 0.93 on digital data, while physical experiments with a Livox Mid 40 sensor achieve a clean accuracy of 0.93 and attack success rate of 0.86. The physical comparison is conducted against IRBA, with LidarFooler achieving a mean attack success improvement of 42.5 percentage points in the evaluated setting.

The results also reveal several limitations. Although LidarFooler achieves effective cross model transfer in many cases, its transfer performance is not consistently superior across all model and dataset combinations. Moreover, the attack success rate decreases substantially under certain defense conditions, indicating that LidarFooler is not uniformly robust against all evaluated defenses. These findings highlight the dependence of physical backdoor effectiveness on the target model, dataset, transfer setting, and defense condition.

The proposed attack further assumes sufficient physical access to place or arrange a trigger within the observable range of the LiDAR sensor, which is more feasible in controlled or semi controlled environments than in unrestricted autonomous driving scenarios. In addition, the current evaluation focuses primarily on unimodal LiDAR point cloud classification, while its effectiveness in multimodal systems remains to be established. Future work will investigate more constrained attacker capabilities, flexible trigger placement, varying trigger to target distances and relative poses, as well as multimodal perception settings, to further improve the understanding of physical backdoor security and defense.

## Figures and Tables

**Table 1:** ACC of clean models on different datasets.

Dataset	PointNet	PointNet++	DGCNN	PCT
ModelNet10	0.91	0.92	0.89	0.90
ModelNet40	0.88	0.88	0.92	0.86
ShapeNetPart	0.93	0.93	0.93	0.93

**Table 2:** Performance of LidarFooler and baseline attacks on ModelNet and ShapeNetPart. Boldface indicates the best result within each row, while the light gray background identifies LidarFooler results.

Dataset	Model	PCBA		PointBA-I		PointBA-O		IRBA		LidarFooler	
		ACC	ASR	ACC	ASR	ACC	ASR	ACC	ASR	ACC	ASR
ModelNet10	PointNet	0.86	0.97	0.90	0.94	0.90	0.90	0.91	0.94	0.92	**0.98**
	PointNet++	0.87	0.99	0.93	0.97	0.90	0.88	0.93	0.91	0.92	0.96
	DGCNN	0.92	0.95	0.94	0.97	0.88	0.87	0.94	0.94	0.91	**0.97**
	PCT	0.82	0.84	0.89	**0.98**	0.90	0.90	0.88	0.84	0.90	**0.98**
ModelNet40	PointNet	0.83	**1.00**	0.86	0.92	0.85	0.75	0.80	0.90	**0.88**	0.83
	PointNet++	0.86	0.95	0.91	0.95	0.89	0.86	0.91	0.91	0.91	0.89
	DGCNN	0.90	**0.98**	0.90	0.95	0.86	0.83	0.91	0.90	0.84	0.84
	PCT	0.81	0.86	0.90	**0.96**	0.90	0.82	0.85	0.75	0.88	0.86
ShapeNetPart	PointNet	**0.98**	**0.97**	0.91	**0.97**	0.91	0.83	**0.98**	0.79	**0.98**	0.96
	PointNet++	0.97	**0.97**	0.91	**0.97**	0.91	0.85	**0.99**	0.86	0.98	**0.97**
	DGCNN	0.98	0.97	0.92	0.96	0.94	0.88	**0.99**	0.89	**0.99**	**0.98**
	PCT	0.93	0.94	**0.95**	**0.97**	0.92	0.82	0.87	0.89	0.93	0.93

**Table 3:** ACC and ASR in the physical domain.

Model	IRBA		LidarFooler	
	ACC	ASR	ACC	ASR
PointNet	0.90	0.33	0.95	0.81
PointNet++	0.92	0.73	0.95	0.86
DGCNN	0.28	0.13	0.97	0.86
PCT	0.93	0.54	0.85	0.90

**Table 4:** Physical control experiments for backdoor attribution.

Model	Training	Targeted ASR		
		Clean Input	Control Object	Optimized Trigger
PointNet	Clean	0.03	0.05	0.08
	Backdoored	0.04	0.09	**0.81 **
PointNet++	Clean	0.02	0.04	0.06
	Backdoored	0.03	0.07	**0.86**
DGCNN	Clean	0.03	0.06	0.09
	Backdoored	0.04	0.10	**0.86**
PCT	Clean	0.02	0.05	0.07
	Backdoored	0.03	0.08	**0.90**
**Mean**	Clean	0.03	0.05	0.08
	Backdoored	0.04	0.09	**0.86**

**Table 5:** Sensitivity of LidarFooler to the number and shape of local triggers under two dataset-model settings.

	Circle		Rectangle		Triangle	
No. of Local Triggers	ACC	ASR	ACC	ASR	ACC	ASR
*(a) ModelNet10 with PointNet *						
1	0.92	0.84	0.92	0.93	0.92	0.80
2	0.91	0.91	0.92	0.91	0.91	0.85
3	0.92	0.97	0.91	0.92	0.92	0.93
4	0.91	0.92	0.91	0.92	0.91	0.90
*(b) ModelNet40 with PointNet++*						
1	0.88	0.78	0.88	0.82	0.88	0.75
2	0.88	0.82	0.88	0.84	0.88	0.80
3	0.88	0.88	0.87	0.89	0.88	0.86
4	0.87	0.86	0.88	0.87	0.87	0.84

**Table 6:** ACC and ASR of LidarFooler for randomly selected target classes. The target classes were randomly selected, and the class indices follow the 0-based labeling convention used in our experiments.

Dataset	$y_{t}=7$			$y_{t}=8$			$y_{t}=9$		
	Class	ACC	ASR	Class	ACC	ASR	Class	ACC	ASR
ModelNet10	sofa	0.91	0.88	table	0.91	0.96	toilet	0.91	0.89
ModelNet40	car	0.88	0.82	chair	0.88	0.84	cone	0.88	0.80
ShapeNetPart	knife	0.97	0.99	lamp	0.98	0.97	laptop	0.96	0.88

**Table 7:** Cross-model transferability of LidarFooler and PCBA.

Surrogate Model	Attack	PointNet		PointNet++		DGCNN		PCT	
		ACC	ASR	ACC	ASR	ACC	ASR	ACC	ASR
PointNet	PCBA	0.87	0.97	0.90	0.95	0.92	1.00	0.84	0.84
	LidarFooler	0.92	0.98	0.92	0.97	0.91	0.97	0.90	0.98
PointNet++	PCBA	0.83	0.98	0.93	0.90	0.88	1.00	0.83	0.91
	LidarFooler	0.91	0.96	0.93	0.92	0.90	0.96	0.90	0.98
DGCNN	PCBA	0.85	0.98	0.91	0.95	0.82	1.00	0.85	0.88
	LidarFooler	0.92	0.86	0.92	0.78	0.90	0.86	0.85	0.85
PCT	PCBA	0.91	1.00	0.82	1.00	0.92	1.00	0.86	0.99
	LidarFooler	0.92	0.95	0.92	0.96	0.92	0.98	0.94	0.91

**Table 8:** ACC and ASR on ModelNet10 under data augmentation, preprocessing, and defense methods. The evaluated settings are grouped into three categories: Augmentation, Preprocessing, and Defense. The LidarFooler results are highlighted with a gray background.

Setting	Method	PCBA		PointBA-I		PointBA-O		IRBA		LidarFooler	
		ACC	ASR	ACC	ASR	ACC	ASR	ACC	ASR	ACC	ASR
**Augmentation**	R	0.28	0.19	0.90	0.93	0.90	0.10	0.26	0.42	0.91	0.77
	R3	0.45	0.10	0.91	0.95	0.89	0.11	0.40	0.39	0.90	0.92
	Scale	0.86	0.98	0.88	0.90	0.88	0.90	0.85	0.83	0.88	0.95
	Translation	0.85	0.94	0.89	0.84	0.90	0.82	0.89	0.76	0.88	0.87
	Dropout	0.87	0.98	0.91	0.94	0.90	0.90	0.91	0.88	0.91	0.95
	Jitter	0.85	0.97	0.90	0.95	0.91	0.92	0.90	0.88	0.92	0.97
**Preprocessing**	SOR	0.93	0.67	0.89	0.62	0.91	0.82	0.84	0.81	0.82	0.85
**Defense**	DUP	0.94	0.12	0.90	0.30	0.90	0.28	0.88	0.16	0.88	0.41
	LPF	0.76	0.77	0.83	0.92	0.83	0.90	0.72	0.80	0.84	0.83

*Notes.* R: $\theta_{y}=90^{\circ }$; R3: $\theta_{x}=\theta_{y}=\theta_{z}=90^{\circ }$ (applied in *x*-*y*-*z* order about fixed axes); Scale: s=0.8; Translation: t=(0.1,0.1,0.1); Dropout: p=0.2; Jitter: σ=0.01, clipping threshold =0.05. Coordinate-dependent parameters are in normalized units.

**Table 9:** Effect of different loss terms on LidarFooler.

$L_{\mathrm{cls}}$	$L_{\mathrm{dist}}$	$L_{\mathrm{center}}$	ASR	CD × 100	WD × 0.1
	✓	✓	0.75	2.28	0.06
✓		✓	0.93	3.66	0.06
✓	✓		0.90	3.65	0.06
✓	✓	✓	0.97	2.27	0.05

## Data Availability

The data and code supporting the findings of this study are available from the corresponding author upon reasonable request.
